# An *in silico *approach combined with *in vivo *experiments enables the identification of a new protein whose overexpression can compensate for specific respiratory defects in *Saccharomyces cerevisiae*

**DOI:** 10.1186/1752-0509-5-173

**Published:** 2011-10-25

**Authors:** Annie Glatigny, Lise Mathieu, Christopher J Herbert, Geneviève Dujardin, Brigitte Meunier, Marie-Hélène Mucchielli-Giorgi

**Affiliations:** 1 CNRS, Centre de Génétique Moléculaire, UPR3404, FRC3115, 91198 Gif-sur-Yvette, France; 2 UVSQ, 55 avenue de Paris, 78035 Versailles, France; 3 Université Pierre et Marie Curie- Paris 6, 75005 Paris, France

## Abstract

**Background:**

The mitochondrial inner membrane contains five large complexes that are essential for oxidative phosphorylation. Although the structure and the catalytic mechanisms of the respiratory complexes have been progressively established, their biogenesis is far from being fully understood. Very few complex III assembly factors have been identified so far. It is probable that more factors are needed for the assembly of a functional complex, but that the genetic approaches used to date have not been able to identify them. We have developed a systems biology approach to identify new factors controlling complex III biogenesis.

**Results:**

We collected all the physical protein-protein interactions (PPI) involving the core subunits, the supernumerary subunits and the assembly factors of complex III and used Cytoscape 2.6.3 and its plugins to construct a network. It was then divided into overlapping and highly interconnected sub-graphs with clusterONE. One sub-graph contained the core and the supernumerary subunits of complex III, it also contained some subunits of complex IV and proteins participating in the assembly of complex IV. This sub-graph was then split with another algorithm into two sub-graphs. The subtraction of these two sub-graphs from the previous sub-graph allowed us to identify a protein of unknown function Usb1p/Ylr132p that interacts with the complex III subunits Qcr2p and Cor1p. We then used genetic and cell biology approaches to investigate the function of Usb1p. Preliminary results indicated that Usb1p is an essential protein with a dual localization in the nucleus and in the mitochondria, and that the over-expression of this protein can compensate for defects in the biogenesis of the respiratory complexes.

**Conclusions:**

Our systems biology approach has highlighted the multiple associations between subunits and assembly factors of complexes III and IV during their biogenesis. In addition, this approach has allowed the identification of a new factor, Usb1p, involved in the biogenesis of respiratory complexes, which could not have been found using classical genetic screens looking for respiratory deficient mutants. Thus, this systems biology approach appears to be a fruitful new way to study the biogenesis of mitochondrial multi-subunit complexes.

## Background

The oxidative phosphorylation system (OXPHOS) is located in the inner membrane of mitochondria and is responsible for the production of ATP, the energy currency of the cell. It is composed of five large complexes, the complexes of the respiratory chain (complexes I to IV) and the ATP synthase, or complex V. The model organism *Saccharomyces cerevisiae *lacks complex I, but instead has three single subunit NADH dehydrogenases. The other OXPHOS complexes II-V are similar to the mammalian complexes. Complex II is composed of four subunits of nuclear origin while the complexes III, IV and V are composed of subunits of dual genetic origin (10, 11 and 19 subunits respectively in *S. cerevisiae*). A few core subunits are encoded by the mitochondrial genome, translated on mitochondrial ribosomes and immediately inserted into the membrane, while most subunits are encoded by nuclear genes, translated in the cytoplasm and imported into mitochondria *via *the TOM/TIM machineries (for a review see [1]). The assembly of the subunits appears to be coordinated (for examples see [2-5]) by site-specific translation mechanisms, both within and at the periphery of the mitochondria and numerous assembly factors that are extrinsic to the complexes have been identified. In addition, supra-complex associations, either between different mitochondrial complexes, or with the TIM import machinery have been reported [6-10]. However, the assembly of the complexes, especially of complex III, and the interactions between the complexes during their assembly is poorly understood.

Complex III occupies a central position in the respiratory chain, between the entry points of the electrons (NADH dehydrogenases and complex II) and their exit, complex IV. The crystallographic structure of the enzyme from several sources has been determined [11-14], and a modular assembly scheme has been proposed ([15] and references within). Until now, only six assembly factors specific for complex III have been reported: Cyt2p, Bcs1p, Cbp3p, Cbp4p, Mzm1p (or Aim8p) and Bca1p [16-19], while more than 25 have been identified for the complex IV (for review see [5]), an enzyme of similar complexity, suggesting that many of the factors involved in complex III biogenesis remain to be discovered.

In order to identify proteins strongly connected with complex III and to look for proteins participating in its assembly process, a systems biology approach could be used. Many high-throughput datasets coming from genetic and proteomic studies concerning the yeast *S. cerevisiae *are accessible *via *public databases [20]. The interactome represents all the physical interactions between proteins of the cell. Two main techniques have been used to produce high quality and complementary data: yeast two-hybrid studies (for examples see [21-24]), which enable systematic pair wise PPI identification and affinity-purification followed by mass spectrometry (AP/MS) (for examples see [25-27]). The integration of all these data generates a network represented by a graph where every node corresponds to a protein and every edge to an interaction. This large network is too complex to be used directly, so specific tools are needed in order to extract the relevant information from this network. Many algorithms and methods of protein network analysis (clustering and assessment of data quality) have been developed and tested on the various datasets available for the yeast *S. cerevisiae*.

Brohée and Van Helden [28] tested different algorithms for clustering PPI networks. Starting from all the PPI data of *S. cerevisiae *they showed that, they could find all the 220 protein complexes presently documented in the MIPS database [29]. Amongst these algorithms, some enable the extraction of independent classes (every protein is a part of a single module), while others enable the extraction of overlapping classes (a protein can be a part of several modules). Each of these algorithms identifies sub-networks having a specific topology: weakly interconnected clusters [30] or strongly interconnected clusters [31]. Within the latter group, MCODE [32] and ClusterONE [33] identify large-sized clusters. This is particularly interesting for our purpose, because biochemical analyses suggest that there are few assembly modules for complex III and that these modules contain numerous strongly interconnected proteins.

In this report, we used a systems biology approach that consists of the integration of data from different types of experiments, but mainly from large-scale studies. Using the APID [34] and BioGRID databases [20], we collected all the physical interactions concerning the core subunits, the supernumerary subunits and the assembly factors of complex III. After the repeated partitioning of the initial network into sub-graphs we identified a group of proteins having few interactions with the main complex III sub-graph. Cell biology and genetic methods were then used to investigate the function of one of the proteins identified by our *in silico *approach.

## Results and Discussion

### Network modelling

In order to find new proteins involved in complex III biogenesis, we used the Cytoscape facilities [35] to combine all the physical interactions concerning complex III (core proteins, supernumerary proteins and assembly factors, see Table 1) from the APID [34] and BioGRID databases [20].

**Table 1:** List of the proteins of complex III (input list)

Complex III	Ordered Locus Name	UniProt ID	SGD Name
**Catalytic core**	YEL024W	P08067	RIP1
	
	YOR065W	P07143	CYT1
	
	Q0105	P00163	COB

**Supernumerary subunits**	YBL045C	P07256	COR1
	
	YPR191W	P07257	QCR2
	
	YFR033C	P00127	QCR6
	
	YDR529C	P00128	QCR7
	
	YJL166W	P08525	QCR8
	
	YGR183C	P22289	QCR9
	
	YHR001W-A	P37299	QCR10

**Assembly factors**	YKL087C	Q00873	CYT2
	
	YDR375C	P32839	BCS1
	
	YPL215W	P21560	CBP3
	
	YGR174C	P37267	CBP4
	
	YLR077W	Q08023	BCA1
	
	YDR493W	Q03429	MZM1

Complex III is defined by three groups of proteins: catalytic core and supernumerary subunits that are part of the structure, and other proteins involved in the assembly of the complex. UniProt ID was used to retrieve data in APID database whereas in BioGRID (or SGD) it was the SGD Name.

**Figure 1:**
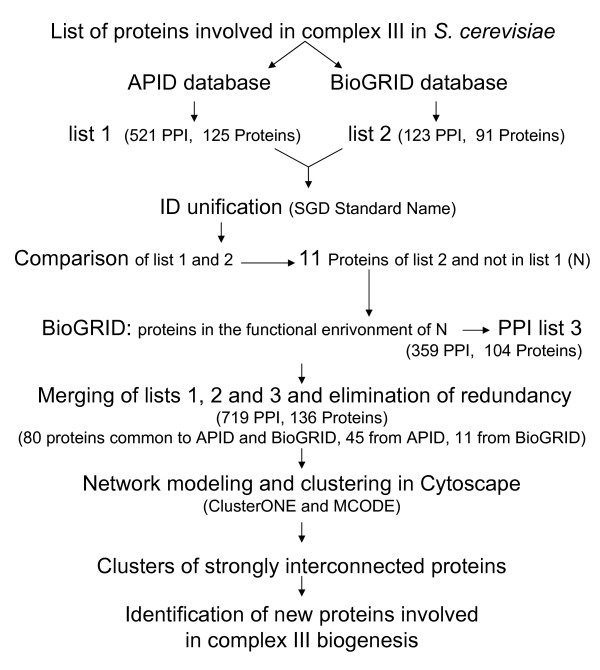
**Computational workflow**. An overview of the network modelling and clustering.

The computational approach leading to the complete PPI network (see section methods) gave the following results:

1) From the APID database, we obtained a list of 521 unique PPI involving 125 proteins (list 1 of Figure 1).

2) From the BioGRID database, 123 unique PPI involving 91 different proteins were collected (list 2 of Figure 1). Some of these interactions are new and allowed us to enrich those obtained from APID.

3) Among these new interactions, some concern eleven new proteins of which some have numerous direct PPI. For example, there are 2645 physical interactions with Nab2p and 1444 with Ubi4p, proteins that are independent of complex III. We will discuss later the consequences of these PPI on the network.

4) The final list, obtained by merging the three PPI lists and eliminating redundancy, was composed of 719 unique PPI involving 136 proteins. 80 proteins are common to both databases, 45 are from APID and 11 from BioGRID. Eliminating redundancy facilitates the identification of links between proteins not often studied and proteins known to be involved in complexes.

5) The resulting network was too large to yield any interesting information. It was necessary to divide it into highly connected sub-graphs that represent functional modules, or protein sub-complexes that we could consider as assembly intermediates.

### Network clustering

To identify new proteins associated with the assembly intermediates of complex III, we looked for sub-groups of strongly interconnected proteins. First we looked for overlapping sub-graphs to take into account the fact that one protein can be associated with several sub-complexes. In this way the network was sub-divided in 26 sub-graphs (clusters 1 to 26), of which six have p-values under 0.05 and good quality scores (Table 2). Note that the clustering method is robust, since the same sub-graphs are found when starting the analysis from only the core and supernumerary proteins.

**Table 2:** Characteristics of the complex III PPI sub-networks obtained with ClusterONE

cluster	nodes	edges	density	quality	p-value
**1**	37	175	0.25	0.476	2.25 10^-7^

**2**	34	157	0.25	0.444	1.01 10^-5^

**3**	36	193	0.29	0.556	1.35 10^-4^

**4**	33	144	0.25	0.414	1.00 10^-3^

**5**	23	101	0.36	0.419	2.00 10^-3^

**6**	16	63	0.450	0.432	0.011

**3.1**	30	185	0.409	0.967	0.000

Cluster 1 is composed of 175 PPI involving 37 proteins; cluster 2 is composed of 157 PPI involving 34 proteins and cluster 4 of 144 PPI involving 33 proteins (Additional file 1 Figure S1). All are built around non-mitochondrial proteins having many interactions independent of complex III. For example Nab2p is a nuclear polyAdenylated RNA-binding protein, Ubi4p is a ubiquitin, Ssa2p is a stress-seventy subfamily A protein, Rpn10p a non-ATPase subunit of the regulatory particle of the 26S proteasome, Tef1p a translational elongation factor and Ssb1p is a ribosome-associated molecular chaperone. Clusters 1, 2 and 4 thus contain PPI that are not specific for complex III.

Cluster 5 is composed of 101 PPI involving 23 proteins and cluster 6 is composed of 63 PPI involving 16 proteins (Additional file 2 Figure S2). The majority of these proteins are translocases involved in the import of proteins into mitochondria, but there are also a few proteins involved in complex III: Bcs1p, Cobp and Cbp2p, a protein of complex IV: Cox7p, and Atp1p and Atp2p, two proteins of the F1 part of complex V. These clusters are clearly not specific for complex III as they include only a few proteins of the input list.

**Figure 2:**
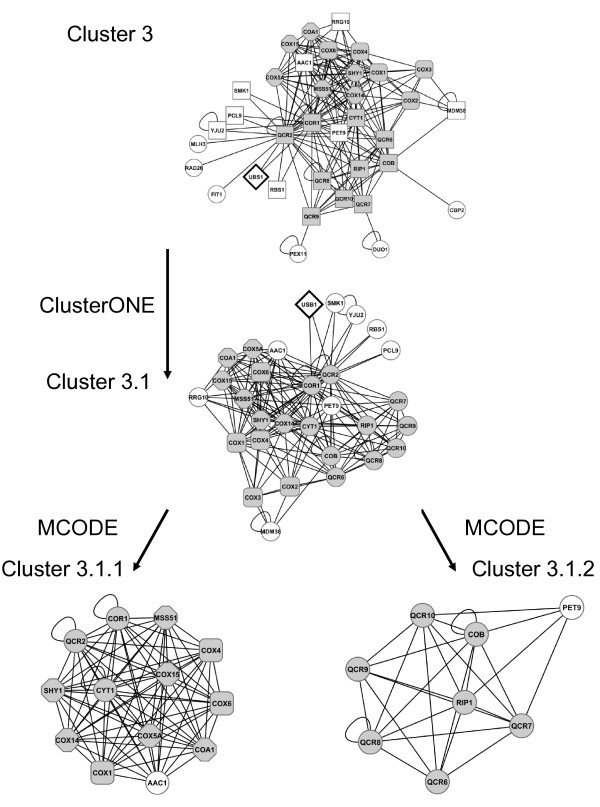
**Complex III sub-graph 3 and its sub-graphs**. Images are taken from Cytoscape. Cluster 3 comes from the partition of the whole PPI network of complex III with ClusterONE. Cluster 3.1 comes from the partition of cluster 3 with ClusterONE. Cluster 3.1.1 and 3.2.2 come from the partition of cluster 3.1 with MCODE. Complex III proteins are represented by grey circles; complex IV proteins by grey rounded squares; complex IV assembly factors by grey octagons; Usb1p by a white lozenge and other proteins by white circles.

Cluster 3 (Figure 2) is composed of 193 PPI involving 36 proteins, including all the complex III subunits. When it was split again into overlapping sub-graphs, only one sub-graph (cluster 3.1) was extracted, with 185 PPI involving 30 proteins. Its quality is equal to 0.97. Most of proteins of this sub-graph are members of complex III (core and supernumerary subunits) and complex IV (Cox1p, Cox2p, Cox3p, Cox4p, Cox5Ap, Cox6p, Cox14p, Cox15p) or participate in the assembly of complex IV (CoA1p, Mss51p, Shy1p, Rrg10p/CoA3p). The fact that this network contains proteins from complexes III and IV, although our input list was exclusively composed of proteins of complex III is probably related to the existence of III-IV super-complexes, which have been clearly identified in blue native gels of mitochondrial extracts [6,7]. Our results show that many associations link these complexes. Furthermore, several assembly factors of complex IV identified here interact with several subunits of complex III: Cor1p, Qcr2p, Cyt1p, suggesting that some factors may play a role in the assembly of both complexes III and IV.

As it was no longer possible to split the cluster 3.1, which is still large, into overlapping sub-graphs it was divided into independent clusters: cluster 3.1.1 is composed of 81 PPI involving 13 proteins belonging to complex IV, three proteins of complex III (Cor1p, Qcr2p and Cyt1p) and Aac1p, a minor isoform of the ADP/ATP mitochondrial transporter (Figure 2). Cluster 3.1.2 is composed of 27 PPI involving 8 proteins; most of them are members of complex III (Cobp, Qcr6p, Qcr7p, Qcr8p, Qcr9p, Qcr10p, Rip1p) and one protein Pet9p/Aac2, the major isoform of the ADP/ATP transporter (Figure 2). Thus the cluster 3.1 can be separated into two parts corresponding broadly to complexes III (see cluster 3.1.2) and IV (see cluster 3.1.1). However, a few proteins are linked with the proteins of the other complex rather than with the proteins of their own complex. For example, Cor1p and Qcr2p are in the sub-graph of complex IV rather than in the sub-graph of complex III as they have more interactions with proteins of complex IV and Aac1p than with the other subunits of complex III. This strengthens the hypothesis of the existence of common factors involved in the assembly process of both complexes.

If we subtract the two sub-clusters (3.1.1 and 3.1.2) from cluster 3.1, nine proteins are left, of which five (Usb1p, Smk1p, Yju2p, Pcl9p, Rbs1p) are connected to Cor1p and Qcr2p. They form a sub-graph, which is not interconnected enough to be detected by MCODE.

Among these proteins Usb1p is the only one whose function is unknown. The association of these three proteins (Cor1p, Qcr2p and Usb1p) corresponds to a complex isolated by tandem affinity purification by Gavin et al. [25]. Thus Usb1p is connected with complex III, and more particularly to the Cor1p [25,26] and Qcr2p sub-units [25,27] (see also Additional file 3 Table S1: the list of all the proteins interacting with Usb1p present in our network). This result obtained *in silico *led us to study the function of Usb1p using genetic and cell biology methods.

### Usb1p is located in both the nucleus and the mitochondria

The *in silico *analysis has shown that Usb1p belongs to the cluster 3.1 that contains all the complex III subunits, six out of the 11 subunits of complex IV, as well as six assembly factors of complex IV. In order to determine if Usb1p could be involved in the biogenesis of the mitochondrial respiratory complexes III and/or IV, we first analyzed its sub-cellular localization.

**Figure 3:**
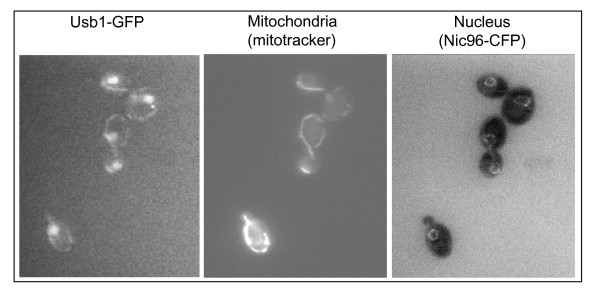
**Dual localization of Usb1p**. Cells expressing the Usb1-GFP and Nic96-CFP fusion proteins were grown in complete glucose medium with a five times excess of adenine at 28°C, washed once and incubated with Mitotracker red CMX ROS (MT, Molecular Probes) for 10 min. After two additional washes, cells were observed by fluorescence microscopy with the appropriate filters to visualize GFP, CFP or Mitotracker.

Large-scale analysis of the localization of GFP-tagged *S. cerevisiae *proteins suggested that Usb1p exhibits a multiple location in the nucleus, the cytoplasm and the mitochondria [36]. To verify this localization, Usb1p was tagged at its C-terminus with GFP. The genetic construction was integrated at the *USB1 *chromosomal locus in a strain carrying a CFP-tagged Nic96 protein that revealed the pores of the nuclear membrane [37]. The *USB1-GFP *gene is therefore expressed under the control of its own promoter sequence in order to avoid mislocalization due to overexpression when the construction is carried on a plasmid. Cells expressing the Nic96-CFP and the Usb1-GFP fusion proteins were examined by fluorescence microscopy using the appropriate filters. Mitotracker was used to stain the mitochondrial network. As shown in Figure 3 andAdditional file 4 (Figure S3), Usb1-GFP was located within the nucleus (3A) whose membrane was also stained by the Nic96-CFP (3C) as well as in fluorescent tubules also stained with the Mitotracker probe (3B). Thus the Usb1-GFP protein has a dual localization in the nucleus and mitochondria.

### Overexpression of *USB1 *compensates specific respiratory defects

**Figure 4:**
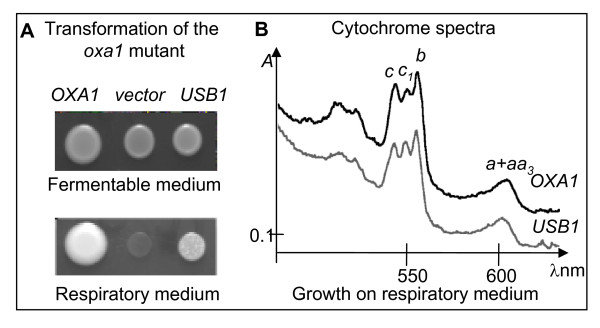
**Overexpression of *USB1 *compensates for a respiratory deficiency**. Panel A: The thermosensitive mutant *oxa1-E65GF229S *[38] was transformed with high-copy plasmids carrying the wild type *OXA1 *gene or the *USB1 *gene, or the empty control vector (vector). The transformants were spotted onto fermentable (glucose) and respiratory (glycerol/ethanol) medium. The plates were incubated for five days at 36°C. Panel B: Cytochrome absorption spectra of the *oxa1 *cells transformed with the high copy plasmids carrying *OXA1 *or *YLR132*, grown on respiratory medium for 2 or 5 days at 36°C. Cytochrome *c1 *and *b *are part of respiratory complex III while cytochromes *a+aa3 *are part of complex IV.

Systematic deletion analysis suggested that the gene could be essential [26]. Indeed, we were not able to obtain any viable haploid cell carrying a deleted allele of the *USB1 *gene from a heterozygous diploid strain (see Methods). To further investigate its role, within mitochondria, we have analyzed the effect of the overexpression of the *USB1 *gene on a set of respiratory deficient mutants. We have cloned *USB1 *on a multicopy plasmid and transformed either a wild type strain or a collection of respiratory deficient mutants that carry mutations in various factors essential for the biogenesis of the respiratory complexes. Table 3 and Figure 4A show that the overexpression of *USB1 *compensates for the respiratory deficiency of an *oxa1-E65G-F229S *mutant and the deletion of *MFT2*, but not a *bcs1 *mutant, or the deletions of *OXA1*, *BCS1 *or *RMD9*. The same level of compensation was observed with the *oxa1 *and *mtf2 *mutants. We have previously shown that the respiratory deficiency of the *oxa1-E65G-F229S *mutant is correlated to a strong decrease of cytochrome *b *of complex III and cytochromes *aa3 *of complex IV [38]. Cytochrome spectra of cells grown on respiratory medium show that the overexpression of *USB1 *in the *oxa1 *mutant restored the assembly of cytochromes *b *and *aa3 *(Figure 4B). Thus the overexpression of *USB1 *specifically compensates for some respiratory deficiencies.

**Table 3:** Overexpression of Ylr132 compensates for specific respiratory deficiencies

Genotype	Transformation with the empty vector	Transformation with the wt gene	Transformation with *YLR132*
*oxa1*	-	++	+

*Δoxa1*	-	++	-

*bcs1*	-	++	-

*Δbcs1*	-	++	-

*Δmtf2*	-	++	+

*Δrmd9*	-	++	-

Respiratory-deficient mutants affecting various steps in the biogenesis of the respiratory complexes were transformed with three different high-copy plasmids: (1) the empty control vector, (2) the plasmid carrying the corresponding wild type gene (*OXA1 *for lines 1 and 2; *BCS1 *for lines 2 and 3; *MTF2 *for line 5 and *RMD9 *for line 6), (3) the plasmid carrying the wild type *USB1 *gene. The transformants were selected on minimal glucose medium lacking uracil to select for the plasmids and their growth were tested on non fermentable medium containing 2% glycerol and 2% ethanol at 28 or 36°C. +: respiratory growth; - no growth. See also Figure 4A. The *oxa1 *mutant carries a double amino-acid substitution E65G-F229S in the Oxa1 protein [38]. The *bcs1 *mutant carries a single amino-acid substitution F342C in Bcs1p [53]. The other mutants carry gene deletions as indicated [38,44,53].

## Conclusions

Although several computational approaches combined with experimental methods (for examples [39,40]) have recently established a comprehensive network for the mitochondrial proteome, our analysis is the first systems biology study strictly devoted to the OXPHOS complexes in yeast. As there are huge amounts of interaction data from different sources (genetic, proteomics) it is not easy to choose which gene/protein is the best candidate for further analysis in a complicated system like OXPHOS, which is composed of several complexes. Thus, an integrative method can help to orient subsequent research and can also highlight specific interactions between the complexes.

Our approach revealed multiple associations between subunits of complexes III and IV and interactions with the ADP/ATP transporters. Cluster 3.1 identifies connections between complex III subunits, complex IV subunits and assembly factors, suggesting some interaction during the assembly process of these two complexes (see [41] and references therein for review on complex IV assembly factors). For example, our results show that the assembly factor Shy1p is connected with all the proteins of the cluster 3.1.1, i.e. with the complex III subunits, Cor1p, Qcr2p, Cyt1p and some subunits and assembly factors of complex IV. The minor ADP/ATP transporter, Aac1p is also part of the same cluster. Cluster 3.1.2 is composed of core (Rip1p, Cobp) and supernumerary proteins (except Cor1p, Qcr2p), as well as Pet9p/Aac2p, the major ADP/ATP transporter.

Although some subunits of the complex II (Sdh3p and Sdh4p) are present in the PPI network we did not retrieve them in the clusters, suggesting that the interactions between complex III and complex II are weak. Cyc1p is also present in the PPI network but there is no strong connection with cytochrome *c*. However, cytochrome *c *is a mobile carrier, shuttling electrons between complexes III and IV and the interactions are probably too dynamic to be detected by our analysis that only detects strongly interconnected proteins. No connections have been found between complex III and the NADH dehydrogenases suggesting that there is no tight contact between them.

Finally, the approach identified a protein Usb1p that had probably escaped previous genetic screens because it is essential for cell survival. This protein has a dual localization, in the nucleus and mitochondria. Overexpression of *USB1 *compensates for the respiratory deficiency of an *oxa1 *mutant. Oxa1p mediates the co-translational insertion of mitochondrially-encoded subunits of the respiratory complexes within the inner membrane (for review see [42]). We have previously shown that an increase in the steady state level of mitochondrial mRNAs encoding respiratory complex subunits can also compensate for the respiratory deficiency of this *oxa1 *mutant *via *the overexpression of *RMD9*. Rmd9p controls the processing/stability of all the mitochondrial mRNAs encoding respiratory subunits but might also control the initiation of mitochondrial translation [38-43]. The overexpression of *USB1 *cannot compensate for the absence of Rmd9p but can restore respiratory growth to a deletion of *MTF2 *(*NAM1*) that affects the processing/stability of two mitochondrial mRNAs, encoding two subunits of complex V [44]. Mtf2p was also shown to interact with the mitochondrial RNA polymerase [45]. Finally, Usb1p was detected in a systematic screen for RNA-binding proteins in yeast [46]. Because of the dual location of Usb1p, in the nucleus and mitochondria, it is tempting to suggest that Usb1p might control the processing or stability of nuclear and mitochondrial RNAs. As the mitochondrial respiratory function is not required for yeast viability, the essential function of Usb1p would be due to its eventual role in stability/processing of nuclear RNA(s).

Thus, the sub-graph analysis that we have developed has highlighted the strong connections between subunits and assembly factors of the complexes III and IV and has led to the identification of a new factor acting in the biogenesis of respiratory complexes in yeast.

## Methods

### Databases

To construct our network we collected data from different sources. Information about protein-protein interactions (PPI) were from APID (Agile Protein Interaction DataAnalyzer) which integrates and unifies PPI from different public repositories [34]. This database includes data from high throughput proteomic techniques as well as small-scale experimental data; data quality is critical as it indicates the reliability of a given interaction. For *S. cerevisiae*, 5902 proteins (from a total of about 6510) and 105728 separate interactions were listed in the last release (February 2009). APID is appropriate for our purpose because it qualifies the functional environment of a given protein. Looking at the neighbours of one protein expands the resulting network and gives a more reliable map thus allowing us to find proteins that could be involved in the same biological process. Unfortunately, the APID database is not updated. So to collect the data of recent experiments we used the BioGRID database (The Biological General Repository of Interaction Datasets), release 3.1.72 (December 25^th^, 2010), which is updated monthly and archives genetic and protein interaction data from model organisms and humans including *S. cerevisiae*. For the budding yeast the complete coverage of the literature is maintained and BioGRID provides interactions to the Saccharomyces Genome Database (SGD). When the data were retrieved, 163188 non-redundant physical and genetic interactions were listed in the base, corresponding to 6049 unique proteins [20]. We eliminated the genetic interactions because not all the genes of complex III proteins were submitted to the high throughput experiments like the Synthetic Genetic Array analysis [47], thus the inclusion of the genetic interactions would lead to a lack of balance in the network. For example there are no interactions for *COR1 *in the Synthetic Genetic Array analysis as it was not included, but there are over one hundred interactions for *QCR2*.

Complementary information was from the SGD [48].

### Network modelling in Cytoscape

Figure 1 shows the computational workflow from the structural and assembly proteins of complex III to the identification of new proteins implicated in complex III biogenesis. The input list of the proteins already known to be involved in complex III is given in Table 1 (core subunits, supernumerary subunits and assembly factors). A five-step approach was necessary to build the complete PPI network:

1) By using the APID2NET plugin of Cytoscape [49] and the UniProtID of the proteins listed in Table 1 (input list), we collected the PPI list: list 1. It corresponds to the direct PPI and to interactions between two proteins having one direct interaction with a protein of the input list. Then, we collected all the physical interactions involving proteins of the same input list from the BioGRID database. We obtained an output list, list 2, composed of PPI involving proteins named by their standard SGD name.

2) To use all the interactions of both APID and BioGRID, we need to fuse the lists but before merging them it is necessary to unify the names. APID and BioGRID do not use the same identifiers (UniProt names and SGD names respectively). We used the SGD names, as they are the standard names used by the yeast community.

3) To be consistent with the APID results we had to look for the functional environment of the new proteins that were present in BioGRID (list 2) and absent in APID (list 1). We compared output lists, list 1 from APID and list 2 from BioGRID, to find proteins that are absent in APID list. We then added all physical interactions between these "new" proteins and the proteins of list 1 and absent from the input list. In this way we obtained the list 3.

4) We then merged the lists 1, 2 and 3, and PPI that were identified by several different experiments were taken into account only once to give a list of unique PPI.

5) This final list was imported into Cytoscape 2.6.3 [35]. The resulting network is un-weighted, since each PPI was kept only once. As it is too large to yield any interesting information it is necessary to divide it into connected sub-networks that might represent functional modules or protein sub-complexes.

The network is un-weighted, i.e. no score is assigned to the edges. When a protein is very well studied, lots of experiments describe its partners and one interaction could be identified several times. As the number of occurrences of an interaction is a criterion of reliability, it could be advantageous to attribute a higher weight to the edges that were the more frequent. However, the weighting would introduce an important bias, as it would favour the most studied proteins.

### Network clustering

To find new proteins associated with assembly intermediates of complex III requires the identification of sub-complexes or assembly modules. In a PPI network, sub-graphs of highly interconnected proteins can be considered as protein complexes or functional modules. Since the same protein can interact with several sub-complexes (this is the case of the assembly factors or chaperons) they must have the possibility of overlapping.

The partition of the resulting PPI network into overlapping sub-graphs of highly interconnected nodes was performed with the plugin ClusterONE (Clustering with Overlapping Neighbourhood Expansion) [33] of Cytoscape. This algorithm works by "growing" dense regions out of small seeds guided by a quality function. The quality of a group is evaluated by the number of internal edges divided by the number of edges involving nodes of the group. Starting from a single seed (in our case: every node) the algorithm extends the group step by step with new edges if they increase the quality of a group. An edge can be removed when its removal increases the quality of its group. The process stops when it is not possible to increase the quality of the groups by adding or removing another edge. Finally, sub-graphs smaller than 3 or having a density (number of edges within the cluster divided by the number of theoretically possible edges), less than 0.25 and a p-value under than 0.05, are discarded and those overlapping significantly (overlap threshold equal 0.8) are merged to form a larger sub-graph; we chose a density of 0.25 to get several overlapping sub-graphs having good quality. Indeed, we suppose that several assembly intermediates having proteins in common are required to make complex III. The p-value of one cluster is computed *a posteriori *with the one-sided Mann-Whitney U test performed on the number of intra edges and external boundary edges. An intra edge is between a cluster node and a node within the cluster. An external boundary edge is between a cluster node and a node outside the cluster.

When a large cluster cannot be further split into overlapping sub-graphs of highly interconnected nodes, it can be split into independent sub-graphs of highly interconnected nodes. The partition into independent sub-graphs was made with the plugin MCODE (Molecular Complex Detection) [32] of Cytoscape. This algorithm detects densely connected regions. First it assigns a weight to each node, corresponding to its local neighbourhood density. Then, it recursively moves outward, including in the cluster the nodes whose weight is above a given threshold. We used MCODE plugin with its default parameters.

### Yeast genetics and biochemical analysis

#### Strains, media, genetic methods, plasmids

CW252 (*mat alpha, ade2, ura3, his3, trp1, leu2*) is the wild type yeast strain. Fermentable media contain 2% glucose while respiratory medium contains 2% glycerol and 2% ethanol. Yeast cells were transformed by the one-step procedure [50]. The high copy vector pFL44 carries the replication origin of the yeast 2 micron plasmid and the *URA3 *marker. YepNB6 contains the wild type *OXA1 *gene and YepML3 the wild type *USB1 *gene; both are cloned in the pFL44 vector.

#### USB1 disruption and epitope tagging of Usb1p

The *USB1 *gene was inactivated in the diploid strain W303, using the PCR method described by Wach *et al*. (1994) [51]: the entire ORF was replaced by the *KanR *marker gene that confers resistance to the drug G418. The heterozygous *USB1*/Δ*usb1::KanR *diploids were sporulated and micro-dissected. Only G418 sensitive spores germinated confirming that the *USB1 *gene is essential. C-terminal epitope tagged proteins were constructed as described: Usb1-GFP using the plasmid pFA6-5GA super bright that uses a G418 resistance marker [52] and Nic96-CFP using pBS4, which carries a hygromycin B resistance marker (The Yeast Resource Center, University of Washington). The strain expressing Usb1-GFP shows growth indistinguishable from the wild type on both fermentable and respiratory medium, showing that the tagged protein is fully functional. The Nic96-CFP strain shows a weak growth defect; because of this the microscopic observations were made in a strain that was homozygous for *USB1-GFP *and heterozygous *nic96-CFP/NIC96*. Fluorescence microscopy was performed using a Zeiss Axioplan 2 microscope linked to a Cool Snap camera (Princeton Instruments).

#### Cytochrome absorption spectra

Cytochrome absorption spectra of whole cells were recorded at liquid nitrogen temperature after reduction by dithionite using a Cary 400 spectrophotometer (Varian, San Fernando, CA). Absorption maxima for the bands of cytochrome *c, c1, b *and *a+aa3 *are expected at 546, 552, 558 and 602 nm, respectively.

## Authors' contributions

AG performed network modelling, network clustering and wrote the manuscript, MHMG conceived all analysis strategies and wrote the manuscript, LM and GD did the genetics and biochemical analysis, CJH did the microscopy analysis, BM conceived the project with MHMG. All authors approved the final version of the manuscript.

## Supplementary Material

Additional file 1 **Figure S1 - Sub-networks 1, 2 and 4 obtained by partition of the whole complex III PPI network with ClusterONE**. Image taken from Cytoscape. Click here for file

Additional file 2 **Figure S2 - Sub-networks 5, 6 obtained by partition of the whole complex III PPI network with ClusterONE**. Image taken from Cytoscape. Click here for file

Additional file 3 **Table S1 - List of Usb1p interactors present in the network **[54]. Click here for file

Additional file 4 **Figure S3 - Localization of Usb1p**. Cells expressing Usb1-GFP were grown in complete medium and stained with Mitotracker red CMX ROS. The figure shows the Usb1-GFP and Mitotracker fluorescent images colored green and red respectively, and the merged image showing the co-localization of the Mitotracker and part of the Usb1-GFP signal. Click here for file
