# Supplementary material for: An in silico approach combined with in vivo experiments enables the identification of a new protein whose overexpression can compensate for specific respiratory defects in Saccharomyces cerevisiae
Source: BMC Syst Biol. 2011 Oct 25;5:173. doi: 10.1186/1752-0509-5-173 (PMC3214889; doi:10.1186/1752-0509-5-173)

Cluster 1
(175 PPI, 37 Proteins)

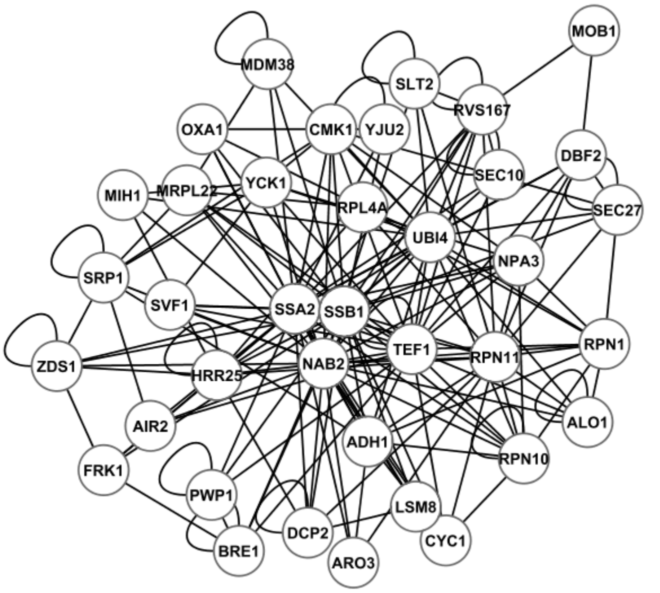

Cluster 2
(157 PPI, 34 Proteins)
Protein from inputlist:
Bca1p

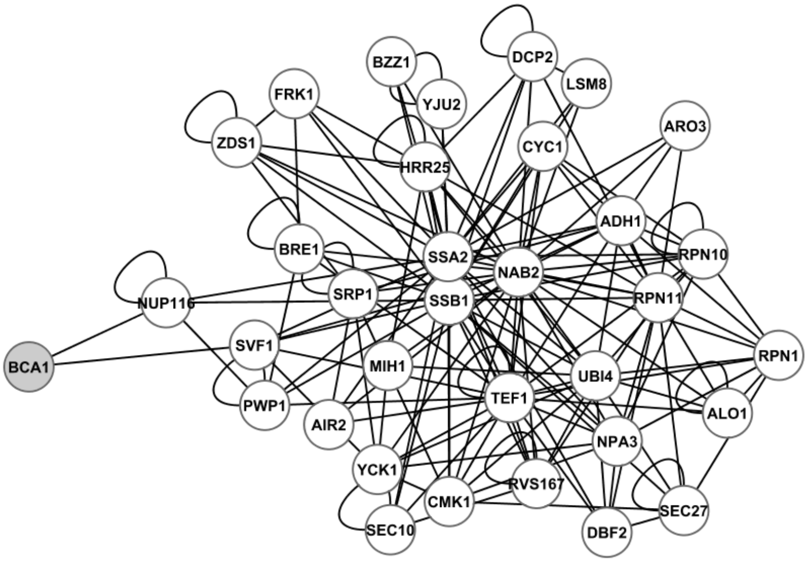

Cluster 4
(144 PPI, 33 Proteins)
Proteins from inputlist:
Cbp3p and Cbp4p

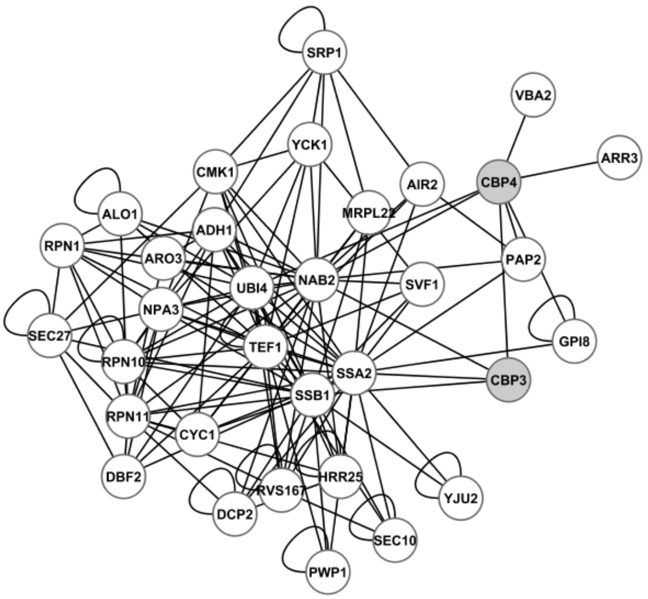

Supplement: Additional file 1 — Figure S1 - Sub-networks 1, 2 and 4 obtained by partition of the whole complex III PPI network with ClusterONE. Image taken from Cytoscape. [file 1752-0509-5-173-S1.PDF]
