# Supplementary material for: An in silico approach combined with in vivo experiments enables the identification of a new protein whose overexpression can compensate for specific respiratory defects in Saccharomyces cerevisiae
Source: BMC Syst Biol. 2011 Oct 25;5:173. doi: 10.1186/1752-0509-5-173 (PMC3214889; doi:10.1186/1752-0509-5-173)

Cluster 5
(101 PPI, 23 Proteins)
Proteins from inputlist:
Bcs1p, Cyt1p

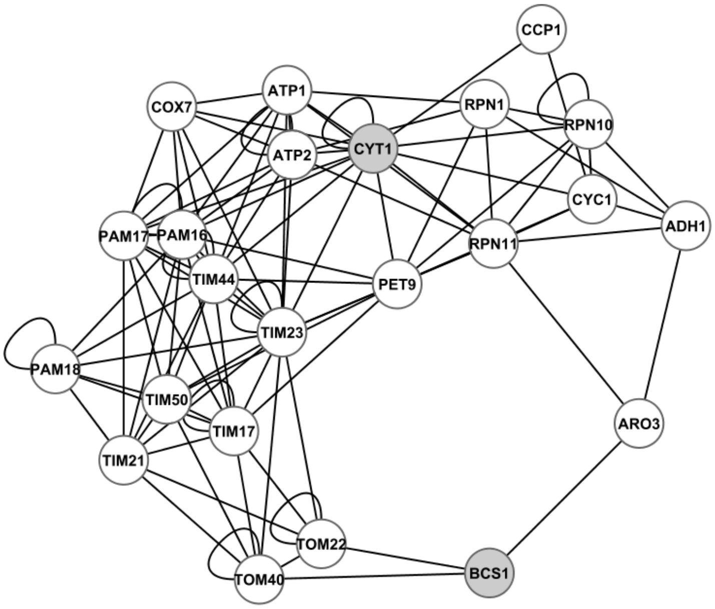

Cluster 6
(63PPI, 16 Proteins)
Proteins from inputlist:
Bcs1p, Cobp

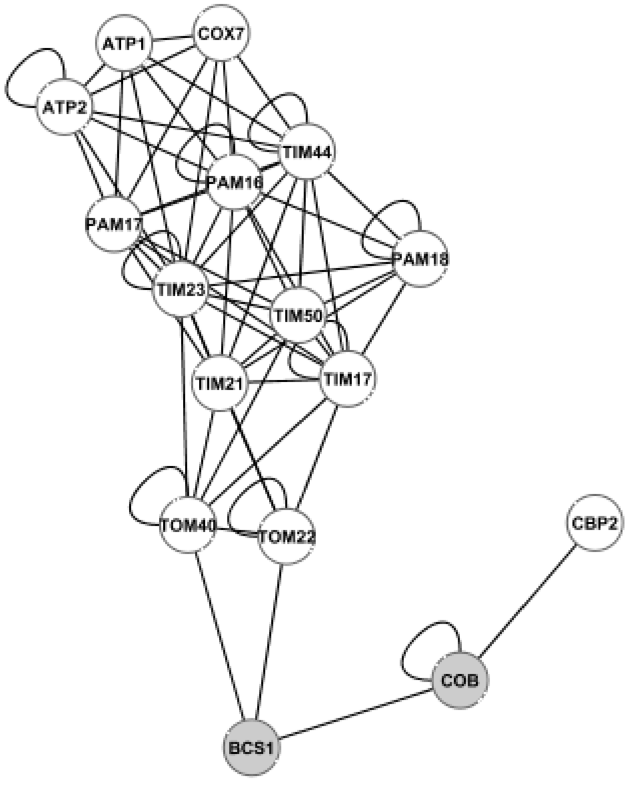

Supplement: Additional file 2 — Figure S2 - Sub-networks 5, 6 obtained by partition of the whole complex III PPI network with ClusterONE. Image taken from Cytoscape. [file 1752-0509-5-173-S2.PDF]
