# Supplementary material for: An in silico approach combined with in vivo experiments enables the identification of a new protein whose overexpression can compensate for specific respiratory defects in Saccharomyces cerevisiae
Source: BMC Syst Biol. 2011 Oct 25;5:173. doi: 10.1186/1752-0509-5-173 (PMC3214889; doi:10.1186/1752-0509-5-173)
Supplement: Additional file 3 — Table S1 - List of Usb1p interactors present in the network [54]. [file 1752-0509-5-173-S3.PDF]

Additional file 4

Table S1 – List of Usb1p interactors present in the network

**SGD Name**	**Ordered locus Name**	**Localization**	**Name description**	**References**
COR1	YBL045C	mitochondrion	subunit of complex IV	[25,26]
QCR2	YPR191W	mitochondrion	subunit of complex IV	[25,27]
TEF1	YPR080W	mitochondrion and cytoplasm	translational elongation factor EF-1 alpha	[25]
YCK1	YHR135C	mitochondrion and plasma membrane	yeast Casein Kinase I homologue	[54]

Among the proteins interacting with Usb1p, 16 in APID database and 14 in BioGRID, only four are present in our network. Note that the interaction with TEF1 is not listed in BioGRID.
