# Supplementary figures and images for: An in silico approach combined with in vivo experiments enables the identification of a new protein whose overexpression can compensate for specific respiratory defects in Saccharomyces cerevisiae
Source: BMC Syst Biol. 2011 Oct 25;5:173. doi: 10.1186/1752-0509-5-173 (PMC3214889; doi:10.1186/1752-0509-5-173)

Usb1-GFP

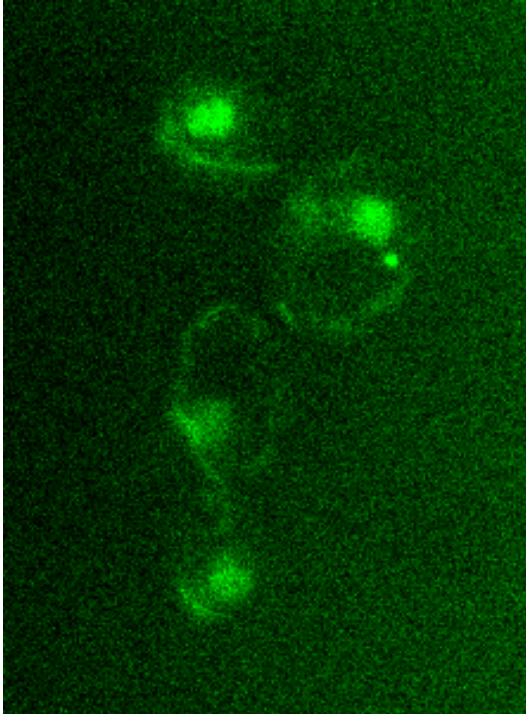

Mitochondria  
(mitotracker)

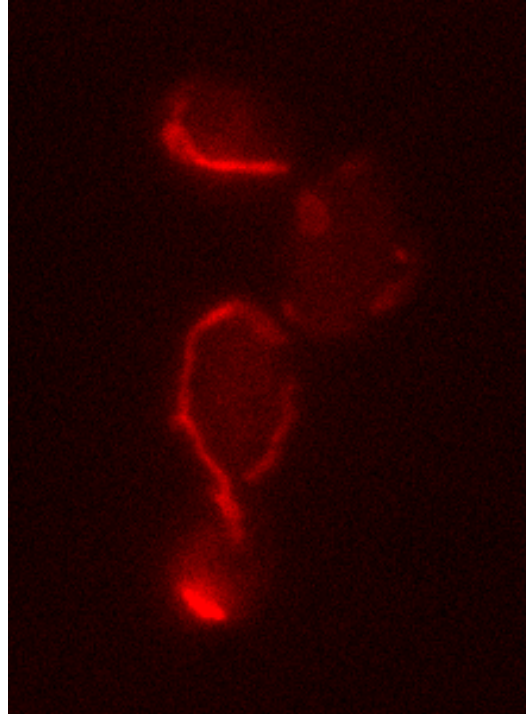

Merge

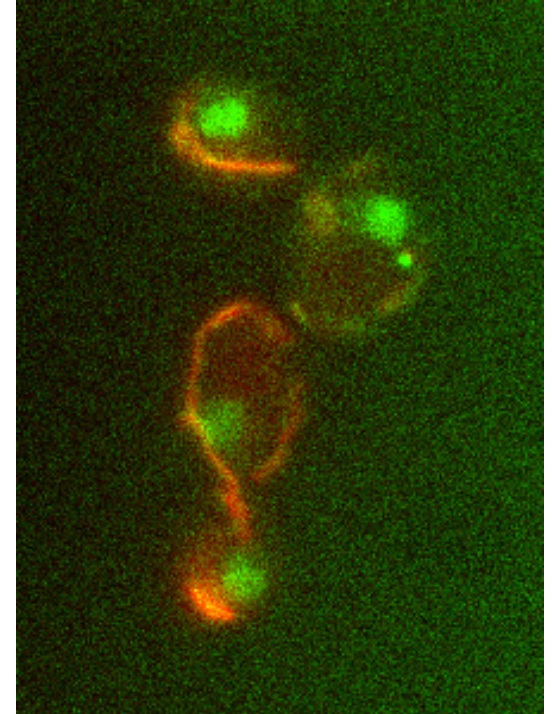

Supplement: Additional file 4 — Figure S3 - Localization of Usb1p. Cells expressing Usb1-GFP were grown in complete medium and stained with Mitotracker red CMX ROS. The figure shows the Usb1-GFP and Mitotracker fluorescent images colored green and red respectively, and the merged image showing the co-localization of the Mitotracker and part of the Usb1-GFP signal. [file 1752-0509-5-173-S4.PDF]
